# New Guidelines for Hypertension Diagnosis and Treatment: An European Perspective

**DOI:** 10.31083/j.rcm2502055

**Published:** 2024-02-04

**Authors:** Guido Grassi

**Affiliations:** ^1^Clinica Medica, Department of Medicine and Surgery, University Milano-Bicocca, 20052 Milan, Italy

**Keywords:** hypertension, guidelines, antihypertensive treatment, cardiovascular risk

Historically, essential hypertension represents one of the first clinical conditions for which diagnostic and therapeutic clinical guidelines have been developed. The first official guidelines paper dates back to 1977 when the Joint National Committee in the United States released the document [1], which was revised few years later in a new paper jointly issued by an “ad hoc” Committee of the World Health Organization (WHO) in conjunction with the International Society of Hypertension (ISH) [2]. During the last decade of the XX century updated versions of the guidelines document have been prepared by the previously mentioned organizations in collaboration with American scientific societies. The first guidelines document prepared by European Societies dates back to 2003, when the European Society of Hypertension (ESH) together with the European Society of Cardiology jointly signed the european guidelines document [3]. Revised editions followed in 2007, 2013 and 2018 [4, 5, 6], the latter being the most cited paper in the worldwide medical literature.

The new guidelines document, released during the 32nd European Meeting on Hypertension held in Milan in 2023 and published in the Journal of Hypertension [7], has been developed by the task force members appointed by the ESH and revised by external reviewers. This editorial will be aimed at briefly discussing the main elements of novelty of the document, whose recommendations are based on three levels of evidence, i.e., those deriving from data collected in randomized clinical trial or meta-analyses (evidence A), in non-randomized clinical trial (evidence B) or in small clinical studies (evidence C) [7].

As in the previous editions ESH Guidelines pay close attention to the clinical value of the different office and out-of-office blood pressure (BP) measurements available in daily clinical practice, namely clinic, home and 24 hour ambulatory values. The “gold-standard” parameter in the diagnostic process of a hypertensive state still remains clinic BP, based on the evidence collected in epidemiological and in controlled intervention trials performed since the early seventies which allowed to define with sphygmomanometric values the relationships between BP and clinical events and their reduction associated with antihypertensive drug treatment (level of evidence A). Guidelines, however, strongly support a wider use of BP measurements obtained outside the clinical environment, i.e., out-of-office, maintaining the same threshods as the 2018 documents [5, 6]. 2023 ESH guidelines document emphasizes the clinical relevance of these out-of-office measurements which are more reproducible and display a greater prognostic value than office BP. Additionally, ambulatory BP allows to detect different clinical hypertensive phenotypes, such as white-coat hypertension, masked hypertension, uncontrolled and controlled high blood pressure, non-dipping, dipping or reverse dipping hypertension, orthostatic hypertension, nighttime hypertension and early morning BP surge [7]. The procedure would also allow to provide information on two additional hemodynamic variables of high clinical and prognostic and clinical relevance, namely BP variability during daytime and nighttime periods and the behaviour of the corresponding heart rate values [7]. Guidelines also emphasize the potential new interest for BP measurement by the patient at his/her home based on the evidence that the approach would allow to counteract one of the barrier to BP control, i.e., poor adherence to antihypertensive drug treatment [8]. Finally, the new guidelines incorporate automated (unattended) BP measurement in the physician’s office [8, 9]. As the document remarks, unattended BP measurement is linked to BP values lower than those obtained by conventional office BP measurement and is likely dependent on the lack of the so-called alerting reaction to the doctor’s BP measurement which may lead, throughout a marked sympathetically-mediated vasocostriction, to a transient visit-related BP elevation [9].

Guidelines, while confirming the relevance for cardiovascular risk stratification of classic risk factors (age, gender, smoking, overweight, obesity, hyperuricemia, elevated heart rate, etc.), add novel variables such as low birth weight, adverse outcomes of pregnancy such as preterm delivery, gestational hypertension and prediabetes, frailty of the older hypertensive patient, impact on BP of the migration processes and environmental exposure to air pollution and traffic noise [7]. ESH guidelines, however, emphasize the need to perform, at any patient’s age [10], risk stratification of the hypertensive patient on the presence of associated clinical conditions (diabetes, heart failure, miocardial infarction, stroke, renal insufficency) and asymptomatic target organ damage. The latter includes several measurements, based on their undisputable predictive value for cardiovascular morbidity and mortality, large availability and cost (electrocardiography, echocardiography, carotid ultrasonography, pulse wave velocity estimated creatinine clearance or glomerular filtration rate through standardized formulae, serum creatinine and microalbuminuria) (level of evidence A) [7]. The 2023 guidelines recommend organ damage to be searched in different organs because of the evidence that multiple organ damage carries a worse prognosis than damage limited to a single organ. They also recommend organ damage to be assessed before and during treatment because data are available that treatment-induced regression of left ventricular hypertrophy and proteinuria reduction are associated with a smaller incidence of cardiovascular events, thereby offering physician and patients an insight on whether the treatment adopted is providing protection (level of evidence A) [7, 11].

New European guidelines confirm clinic BP values amounting to 140/90 mmHg as those above which the therapeutic intervention is needed, with the goal to achieve during treatment values close to 130/80 mmHg. An exception is represented by older patients, in which BP tresholds are 160/90 mmHg and BP goals not below the value of 130/80 mmHg or, better, 135/85 mmHg. To achieve the above mentioned BP goal, along with life-style interventions [12], all 5 classes of antihypertenisve drugs (diuretics, ACE-inhibitors, calcium antagonists, beta-blockers and angiotensin II receptor antagonists) can be used (level of evidence A) [7]. Thus, at variance from the previous guidelines, the 2023 document fully restores the therapeutic role of beta-blocking agents in hypertension treatment, based on the evidence obtained in the context of clinical trials and meta-analyses [13, 14].

Fixed low-dose combination drug therapy represents the first choice to start treatment (level of evidence A). This intervention, according to the recommendations of the guidelines, allows an adequate blood pressure control to be obtained in a short time period, optimizing the patient’s adherence profile to therapy with the use of a reduced pharmacological dosage. The observation made in several clinical intervention studies that adherence to therapy is greater in treatment schemes that include fixed low-dose combination therapy as the first therapeutic step is the ground for this recommendation [7]. The therapeutic choice of the pharmacological association to be followed in the individual patient depends on a number of factors, including the presence of comorbidities, organ damage, patient’s age and metabolic profile, as well as on the detection of a more or less severe global cardiovascular risk profile.

Three other novel aspects of the guidelines document deserve a brief comment. First in the 2023 document the evidences on the clinical usefulness of renal nerves ablation are classified as level of evidence A. This is based on the positive results of recent trials which, by using sophisticated devices to perform bilateral renal denervation, have shown in resistant hypertenive state small but significant ambulatory and office BP reductions, even when the results were adjusted for those obtained in the placebo-control group [15, 16]. Guidelines however, emphasize the need to consider the procedure only in selected clinical conditions, such as in difficult to control and in drug-resistant hypertension, thereby not supporting a wider and generalized use of the approach in a larger fraction of the hypertensive phenotypes.

The second aspect of the guidelines refers to the procedures to be adopted for patients follow-up. A new consideration introduced in these guidelines is the key role of nurses and pharmacists in the long-term treatment of high BP. These professionals have an important role to play in patient instruction, support, and follow-up as part of a general strategy to improve BP control and achieve better adherence to treatment. As far as the timetable of the follow-up, guidelines recommend a regular scheme of visits, to be performed every 3 months initilly and subsequently every year. Optimal adherence to therapeutic intervention, effective BP control and possibly regression of target organ damage represent the goals of the follow-up intervention.

Finally guidelines fully encourage the systematic use in the years to come of the telemedicine appraoch, by allowing to obtain a better adherence of the hypertensive patients to the antihypertensive treatment, thus improving BP control [17].

In this commentary on the new ESH guidelines on hypertension, the attempt has been made to highlight the most important novelties in the document, which is even more extensive than the 2018 edition. Main novelties are multiple and include, amomg others, thersholds and targets of the BP lowering intervention, the early use of combination drug treatment with the aim of improving adherence, BP control and ultimately cardiovascular protection. A larger use of new cardiovascular drugs, such ad sodium glucose transporter-2 inhibitors (SGLT2 inhibitors) and angiotensin receptor neprylisin inhibitors (ARNI), will hopefully allow in the next future to help doctor to achieve this goal.
